# An open-label prospective pilot trial of nucleus accumbens deep brain stimulation for children with autism spectrum disorder and severe, refractory self-injurious behavior: study protocol

**DOI:** 10.1186/s40814-022-00988-3

**Published:** 2022-02-02

**Authors:** Han Yan, Lauren Siegel, Sara Breitbart, Carolina Gorodetsky, Alfonso Fasano, Aliya Rahim, Alvin Loh, Abhaya V. Kulkarni, George M. Ibrahim

**Affiliations:** 1 Division of Neurosurgery, The Hospital for Sick Children, 555 University Avenue, Room 1503, Toronto, ON M5G 1X8 Canada; 2 Institute of Health of Health Policy, Management and Evaluation, University of Toronto, Toronto, Ontario Canada; 3 Division of Neurosurgery, Department of Surgery, University of Toronto, Toronto, Canada; 4 Neurosciences and Mental Health Program, The Hospital for Sick Children, 555 University Avenue, Room 1503, Toronto, ON M5G 1X8 Canada; 5 Division of Neurology, The Hospital for Sick Children, Toronto, Canada; 6 Edmond J. Safra Program in Parkinson’s Disease, Morton and Gloria Shulman Movement Disorders Clinic, Toronto Western Hospital, UHN, Toronto, Ontario, Canada. Division of Neurology, University of Toronto, Toronto, Ontario Canada; 7 Krembil Brain Institute, Toronto, Ontario Canada; 8 CenteR for Advancing Neurotechnological Innovation to Application (CRANIA), Toronto, ON Canada; 9 Surrey Place, Toronto, Ontario Canada; 10 Division of Developmental Paediatrics, Department of Paediatrics, University of Toronto, Toronto, Canada; 11 Institute of Biomedical Engineering, University of Toronto, Toronto, Canada; 12 Institute of Medical Science, University of Toronto, Toronto, Canada

**Keywords:** Autism spectrum disorder, Deep brain stimulation, Children, Self-injurious behavior, Nucleus accumbens

## Abstract

**Background:**

Children and youth with autism spectrum disorder (ASD) may manifest self-injurious behaviors (SIB) that may become severe and refractory with limited pharmacologic or behavioral treatment options. Here, we present the protocol of a prospective, mixed-methods study to assess the safety and efficacy of deep brain stimulation (DBS) of the nucleus accumbens (NAcc) for children and youth with ASD and severe, refractory SIB.

**Methods:**

This is a prospective, single-center, single-cohort, open-label, non-randomized pilot trial of 6 patients. Participants will be recruited through specialized behavioral clinics with persistent severe and refractory SIB following standard and intensive interventions. Following NAcc-DBS, participants will be enrolled in the study for 12 months. The primary objectives of the study are safety and feasibility, assessed by rate of recruitment and identification of factors impacting adherence to follow-up and study protocol. Potential treatment efficacy will be assessed by changes in the Children’s Yale-Brown Obsessive-Compulsive Scale in ASD (CYBOCS-ASD), the Behavior Problems Index (BPI), the Inventory of Statements about Self-Injury (ISAS) and the Repetitive Behavior Scale-Revised (RBS-R) questionnaires. Additional clinical outcomes will be assessed, including measures of participant and caregiver quality of life, actigraph measurements, and positron emission tomography (PET) changes following DBS.

**Discussion:**

This study will be the first to evaluate the effect of DBS of the NAcc on a pediatric population in a controlled, prospective trial. Secondary outcomes will improve the understanding of behavioral, neuro-imaging, and electrophysiologic changes in children with ASD and SIB treated with DBS. This trial will provide an estimated effect size of NAcc-DBS for severe refractory SIB in children with ASD in preparation for future comparative trials.

**Trial registration:**

Registration on ClinicalTrials.gov was completed on 12 June 2019 with the Identifier: NCT03982888.

## Background

Autism spectrum disorder (ASD) is a clinical diagnosis based on a set of heterogeneous neurodevelopmental conditions. ASD is characterized by difficulties in social interaction, communication, repetitive restricted behaviors according to the Diagnostic and Statistical Manual of Mental Disorders, 5th edition (DSM-V) [1]. ASD has a worldwide prevalence of 1%, an estimated 1 in 68 births in the USA in 2014 by the Center for Disease Control and Prevention, and a greater prevalence in males than females [2–4].

The behavioral comorbidities of ASD include aggressive behavior (up to 68%) [5, 6] and repetitive self-injurious behavior (up to 50%) [7, 8]. In children with ASD and self-injurious behaviors (SIB), over 75% of children will have persistence of these behaviors into adulthood, sometimes resulting in severe harm and even death [5, 9–11]. SIB can be defined as repetitive “behavior which produces physical injury to the individual’s own body” with several different subclassifications [12]. Pharmacologic agents, such as antipsychotics (e.g., risperidone, aripiprazole), and selective serotonin reuptake inhibitors (e.g., fluoxetine) show evidence for treating irritability but not specifically reducing self-injury [13–17].

Applied behavior analysis, based on operant methodology, offers an evidence-based method to assess and treat SIB. Functional analysis identifies the function of the behavior and shows what conditions self-injury are associated with and what stimulus conditions maintain it [18]. It is hypothesized that social consequences, such as escape from demands or selective attention, mediate 20–25% of SIB through automatic reinforcement [12, 19]. Subtypes of automatic reinforcement show differential responses to reinforcement-based interventions and may have implications on the biological bases of SIB and biobehavioral research. This may also give us insight into the role of neuromodulation as a feasible intervention for SIB.

Neuromodulation, through deep brain stimulation (DBS), may present novel treatment options for this population when other treatments are not effective or tolerated. DBS is a precise neuromodulation strategy for targeting pathological brain circuitry [20], albeit with incompletely understood mechanisms of action. DBS involves implantation of electrodes (often two with one on each side of the brain) into deep brain targets and delivery of an electrical current through these electrodes via an impulse generator implanted in the chest. The indication for DBS in pediatric populations is primarily for dystonia, notably inherited dystonia without nervous system pathology [21]. In children with refractory disease processes, DBS has been previously utilized for novel indications, such as select cases of Tourette’s syndrome, obsessive-compulsive disorder (OCD), and epilepsy with varying levels of success [22–26]. In the course of DBS treatment for dystonia and Tourette’s syndrome in adult patients with comorbid SIB, reduction in the frequency or full cessation of SIB has been reported [27, 28]. DBS of several targets has previously been employed to treat SIB, including in six patients under the age of 20 with ASD (Table 1). These targets include the basolateral amygdala [30], globus pallidus internus [31], posterior hypothalamus [32], and the nucleus accumbens (NAcc) [34].

**Table 1:** Literature review of DBS for the treatment of SIB and ASD

[29] Author	Age, sex	Behavior	DBS target (programming)	Pre-DBS score	Post-DBS score
Sturm 2012 [30]	13M	Self-aggression	Basolateral amygdala (120 μs, 130 Hz, 2–6.5 V)	Restraints do not prevent skin lesions and life threatening self-injury	Restraint of the wrists suffices and is well tolerated
Stocco 2014 [31]	19F	Self-picking	Globus pallidus internus (120 μs, 80 Hz, 3.3 V)	JHMRS 46	JHMRS 4
Stocco 2014 [31]	17M	Punching of arms and legs, biting	Globus pallidus internus (120 μs, 100 Hz, 2.5 V) + Anterior limb of internal capsule (210 μs 100 Hz 2.0 V)	JHMRS 67	JHMRS 19
Benedetti-Isaac 2015 [32]	27M	Aggressive behavior towards self	Posterior hypothalamus (90 μs, 185 Hz, 2.7 V)	OAS 9	OAS 1
Benedetti-Isaac 2015 [32]	16M	Self-aggression	Posterior hypothalamus (90 μs, 185 Hz, 2.8 V)	OAS 8	OAS 8 (temporary improvement at 1 month)
Segar 2015 [33]	24F	Biting hands, picking skin	Nucleus accumbens (90 μs, 130 Hz, 8 V)	GAF 20	GAF 50-60
Park 2016 [34]	13M	Self-mutilation, face-hitting causing fractures	Nucleus accumbens (90 μs, 130 Hz, 3–5 V)	CGI-S 6 ABC 106 CY-BOCS 22 K-ARS 54 SRS 101	CGI-S 4 ABC 40 CY-BOCS 7 K-ARS 36 SRS 98
Kakko 2019 [29]	19M	Aggression, self-mutilation, lacerations	Globus pallidus internus		Self-destructive behavior ceased
Doshi 2019 [35]	42F	Hitting, violent outbursts	Nucleus accumbens (60 μs, 130 Hz, 2.6 V)	YBOCS 19 HDS 20 HAS 30 SCQ 26	YBOCS 5 HDS 15 HAS 18 SCQ 16

*ABC* antecedent behavior consequence, *CGI-S* Clinical Global Impairment-Severity, *CY-BOCS* Children’s Yale-Brown Obsessive Compulsive Scale, *DBS* deep brain stimulation, *GAF* global assessment of functioning, *HAS* Hamilton Anxiety Scale, *HDS* Hamilton Depression Scale, *JHMRS* John’s Hopkins motor stereotypy rating scale, *K-ARS* Korean ADHD Rating Scale, *OAS* Overt Aggression Scale, *SCQ* Social Communication Questionnaire, *SRS* Social Responsiveness Scale

The NAcc of the ventral striatum receives projections from the orbitofrontal cortex and sends hierarchical information via spiraling striatonigrostriatal projections to the dorsal striatum [36, 37]. Volumetric studies of the striatum suggest that an imbalance of the ventral-dorsal striatal circuity may underlie SIB [38, 39]. Likewise, the amygdala, a previous target for SIB in children also projects widely to the striatum, with weaker connectivity patterns in children with ASD [40]. The NAcc also possesses widespread projections to the dopaminergic receptors of the ventral tegmental area [36]. Other pathologies, such as OCD, addiction, and alcoholism, have been treated with NAcc-DBS [41–44]. When the NAcc is targeted for DBS, the anterior limb of the internal capsule (ALIC) can be stimulated simultaneously to augment behavioral changes related to attention and sensorimotor control.

### Study design

We present a protocol for a single-center, single-cohort, open-label, non-randomized prospective pilot trial of NAcc-DBS for refractory and severe SIB in children with ASD. The trial is currently open for recruitment at the Hospital for Sick Children (HSC) in Toronto.

### Goals and objectives

The primary objective of the study is to assess feasibility and safety, measured by recruitment rate, identification of factors impacting follow-up and protocol adherence, and successful implantation. Secondary clinical objectives include change in frequency or severity of SIB following surgery, measured regularly by caregiver-reported questionnaires over the course of 1 year. Additional clinical outcomes include changes quality of life for the patient and their caregiver(s), repetitive motion characteristics as measured with actigraphy, metabolic changes seen through positron emission tomography (PET), and changes on electrophysiologic data. To our knowledge, this is the first prospective trial of DBS for children with SIB and represents the first assessment of a potential surgical treatment for patients with extremely limited therapeutic options.

## Methods/design

This is a prospective, single-center, open-label, non-randomized study of six patients at HSC. The aim is to evaluate the feasibility and safety of DBS of the NAcc for medically refractory, repetitive self-injurious behaviors in children with ASD. Electrophysiological parameters are optimized in the 12-month follow-up to study and manage possible side effects.

### Ethics and registry

This research involves human participants and is performed in accordance with the Delcaration of Helsinki. This trial was approved by the Hospital for Sick Children Research Ethics Board, approval number 1000060282. Registration on ClinicalTrials.gov was completed in June of 2019 with the Identifier: NCT03982888. Patient recruitment and continuation in the study is considered by a safety monitoring board at HSC. Written informed consent to participate in the trial is acquired from the child or adolescent when able, or the parent or legal guardians. If the child or adolescent is unable to provide consent, assent is sought.

### Enrollment and surgery

Potential participants are recruited from specialized behavioral clinics. Potential participants are reviewed by the investigators with respect to inclusion and exclusion criteria (Table 2). Eligible patients are discussed in a multidisciplinary meeting of the surgical, psychiatric and medical teams. There are no changes to the participants’ medications or behavior therapies after enrollment prior to surgery. Any changes to medications following surgery are noted, including changes to dosages and indication for change.

**Table 2:** Study inclusion and exclusion criteria

*Inclusion criteria*	
1. Children and adolescents between age 7-18	
2. Diagnosed with autism spectrum disorder by a treating developmental pediatrician	
3. Failure or non-eligibility of medical therapy with ongoing repetitive self-injurious behaviors, at 6 months or more after instigation of therapy. Failure is defined as a lack of improvement in self-injurious behaviors, as documented by objective evidence, including caregiver logs or clinician assessment, if the clinician has documented a baseline status prior to instigation of the medical therapy.	
4. The child has undergone rigorous, gold-standard Functional Behavior Assessment including functional analysis, leading to treatment lasting a minimum of 6 months, without significant change from baseline.	
5. Diagnosis of secondary stereotypies, based on clinical assessment of the treating physicians with evidence of self-injury, documented in the patient records. The definition of self-injury is contextual, but requires current, previous or potential manifestation of physical injury to the child.	
6. The child is at risk of permanent injury as a result of self-injurious behaviors. Permanent injury is non-reversible physical injury causing a reduction in baseline functions.	
7. Parents or legal guardians, including caregivers, informed, and able to give written consent.	
8. Able to comply with all testing, follow-ups and study appointments and protocols for 12 months following the end of the duration of the study.	
*Exclusion criteria*	
1. Does not meet study specific definition of autism spectrum disorder using DSM-5 criteria. The treating physicians at each individual clinic will be responsible for diagnosis.	
2. Substance dependence or abuse in the last 6 months, excluding caffeine and nicotine	
3. Any contraindication to MRI, required for stereotactic surgical planning.	
4. Likely to relocate away from the study site or move during the study’s 1-year duration	
5. Presence of cardiac arrhythmias, coagulopathy or other cardiac, respiratory, renal, or endocrine conditions that will result in significant risk from a surgical procedure.	
6. Pregnancy	

**Fig. 1:**
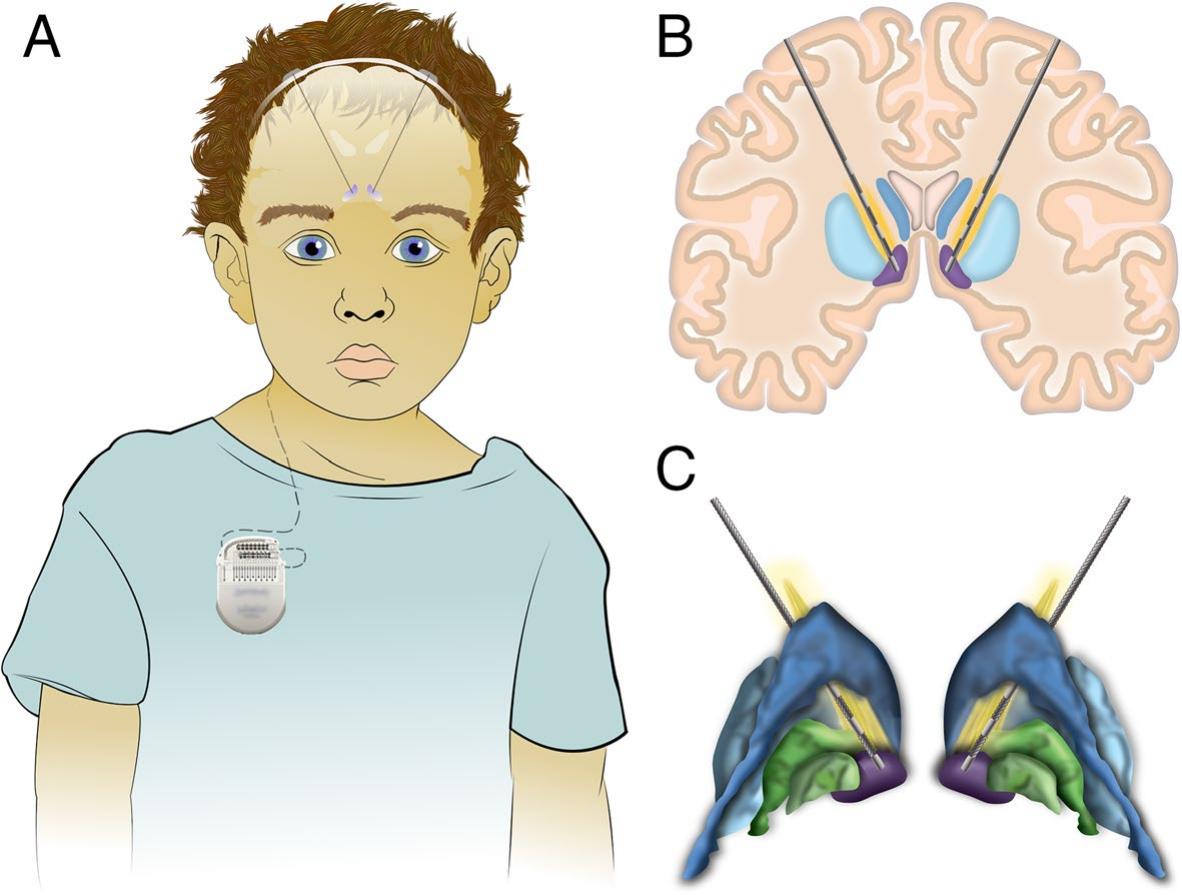
DBS for children, targeting the NAcc. **A** DBS extension wires for children are placed with greater length to allow for growth. **B** Coronal representation of DBS lead trajectory, passing through the ALIC and targeting the NAcc. **C** 3D representation of DBS lead trajectory. NAcc (purple), ALIC (yellow), caudate (dark blue), putamen (light blue), and globus pallidus (green)

Participants are assessed by the anesthesia service, neuropsychology team, and receive a magnetic resonance image (MRI) and optional fluorodeoxyglucose (FDG) PET scan. On the day of surgery, a Leksell stereotactic frame is applied under general anesthesia. The patient receives a head computed tomography (CT) scan that is merged to the pre-operative MRI and planned targets. Stereotactic coordinates are calculated and verified to target the NAcc with the goal to include the ALIC in the trajectory of the DBS electrode (Fig. 1 B, C). In the operating room, a curvilinear incision anterior to the coronal suture is made to place the DBS electrodes stereotactically. Thresholds for side effects are recorded intraoperatively. Electrodes are placed with fluoroscopic confirmation and a pulse generator is connected and implanted in the infraclavicular region under the same anesthetic.

Patients undergo a postoperative MRI to confirm the position of the electrodes. After the surgery, they are monitored in the hospital for 3–5 days and discharged with the stimulator off. Patients start DBS programming at 4–6 weeks after surgery and visit the joint neurosurgery, neurology DBS clinic at HSC. There is regular monitoring for the development of behavioral, motor, and psychiatric effects from stimulation. During weeks 2–6, there are weekly follow-ups to address any potential complications. The full schedule of events is presented in Table 3.

**Table 3:** Follow-up schedule for trial participan ts

		Study period							
		Enrollment	Surgery	Follow-up					Post-trial
Timepoint		Week 8 ± 4 weeks	Baseline W0	Weekly (W1–6) ± 1 day	Every 4 W (W8–56) ± 3 days	Week 12 ± 1 week	Week 26 ± 2 weeks	Week 52 ± 2 weeks	W > 52
Enrollment	Eligibility screen	X							
	Informed consent for trial	X							
	Demographics questionnaire	X							
	Anesthetic evaluation	**X**							
	Informed surgical consent	**X**							
In-hospital treatment and care	Deep brain stimulation leads and IPG insertion		**X**						
	Programming				**X**	**X**	**X**	**X**	**Every 6 months or as indicated**
Imaging	MRI	**X (or earlier)**	**X**						
	CT		**X**						
	FDG-PET (optional)	X						X	
Questionnaires	Children’s Yale-Brown Obsessive-Compulsive Scale in ASD (CYBOCS-ASD)	X		X (week 4)		X	X	X	
	Inventory of Statements about Self-Injury (ISAS)	X		X (week 4)		X	X	X	
	Behavior problems inventory (BPI)	X		X (week 4)		X	X	X	
	Repetitive Behavior Scale-Revised (RBS-R)	X		X (week 4)		X	X	X	
	Quality of Life Scale 9Peds QL	X		X (week 4)		X	X	X	
	Aberrant Behavior Checklist	X		X (week 4)		X	X	X	
Other assessments	Neurosurgery evaluation			X (virtual)	X (virtual)	**X**	**X**	**X**	**Annual follow-up**
	Neuropsychology evaluation	**X**					X	X	As needed
	Actigraphy (optional)	X				X		X	
	Self-injury log	X		X	X	X	X	X	

Bolded cells show standard of care for all pediatric DBS patients

### Sample size and recruitment

In this prospective, controlled trial, six patients are selected to determine safety and initial potential efficacy, as it typical for most DBS early phase trials. There is no control group, and each patient will serve as a comparator for primary outcome measures.

### Feasibility and safety

The trial will be determined feasible if there is > 50% recruitment rate or recruitment of 6 patients in under 36 months as a threshold for progression to a definitive trial. Recruitment rate is measured as the ratio of participants enrolled compared to those who are eligible. New DBS targets typically include 6 patients in pilot trials [45, 46] to establish an initial understanding of effect of therapy through multiple outcomes: clinical, radiographic, and electrophysiologic. There will be additional secondary feasibility outcomes with projected thresholds to determine achievability. These include the proportion of patients who received surgery following enrollment (80%), proportion of patients with full completion of baseline questionnaires (90%), proportion of patients with full completion of multi-modal outcome assessments (i.e., PET, actigraphy) (50%), and percent completion of all clinical outcome data (80%). Families unable to speak English will complete questionnaires through the aid of translators and their completion rate of outcome measures will be compared to English speakers. These data will provide additional information regarding decision-making of progression to a large trial. Any deviance to study protocol or inability to complete clinical questionnaires will be documented with an attempt to understand patient barriers.

The measure of safety outcomes is critical as this routine procedure is being implemented in a new patient population for a novel indication. Immediately peri-operatively in hospital, neurologic complications from surgery (bleeding, seizure) will be monitored and recorded. Longitudinal safety of the intervention will be carefully monitored with weekly phone calls to participants for the first 6 weeks. Participants will be monitored for post-operative complications (i.e., hardware malfunction, signs of infection, unexpected neurologic symptoms) and worsening of severity or frequency of self-injurious behaviors. The expected complication rate is < 10%.

There is no official Data and Safety Monitoring Board (DSMB) for this pilot. Instead, a safety subcommittee consisting of a neurosurgeon, a neurologist, and a developmental pediatrician will meet twice annually to discuss possible safety concerns. This committee would help with the design of the future definitive trial based on the safety concerns raised during this pilot. There are no current stopping criteria outlined, but the safety subcommittee would have the authority to determine cessation of the trial due to unexpected safety concerns.

### Clinical outcome measures

This trial will conduct pre-post analyses to compare frequency and severity of SIB in children and youth with ASD before and after DBS intervention. Clinical outcomes are measured by the change between baseline and 1 year in the following validated scales: Children’s Yale-Brown Obsessive-Compulsive Scale in Autism Spectrum Disorder (CYBOCS-ASD) [47, 48], the Behavior Problems Inventory (BPI) [49], the Inventory of Statements about Self-Injury (ISAS) [50] and the Repetitive Behavior Scale-Revised (RBS-R) [51]. These scales were chosen by expert consensus between developmental pediatricians, neurologists, and the neurosurgical team. Although no scale has specifically been validated for children with ASD and SIB, the combination of these scales studied in children with ASD or all-age patients with repetitive behavior will provide an assessment of responsiveness of DBS over time. Secondary clinical outcomes are measured using the Quality of Life Scale 9Peds QL version 4, Aberrant Behavior Checklist [52, 53], and caregiver logs of repetitive self-injurious behaviors. Time to complete each questionnaire and correlation with quality of life will be useful information in the design of a composite primary objective in larger, future trials.

Patients and caregivers complete scales prior to the intervention and also at week 4, 12, week 26, and week 52. Baseline assessments are completed prior to surgery. Efficacy is determined by the within-subject percent change in the primary outcome scores at 1 year (week 52) following surgery compared to baseline.

Additional outcomes include changes in motor patterns based on actigraphy. Actigraphy is the continuous measurement of an individuals’ movement using a device worn similarly to a watch (Axivity AX3 and Axivity AX6) [54, 55]. The Axivity device can quantify movements and has been validated to detect specific behaviors, such as hand flapping, of patients with ASD with 94.6% accuracy [56]. Children have an actigraph placed on their non-dominant wrist 4 weeks prior to the surgery for a total of 2 weeks of recordings. During specific follow-up visits (weeks 12, 26, and 52), the children have the option to wear the actigraph for 2-week intervals. Markers of stereotypies include maximum and minimum value amplitudes, variance, peak to peak, and entropy fast Fourier transform, to be analyzed using MATLAB (Mathworks, Natick, MA) [54, 56].

Children enrolled in the trial may receive a FDG-PET scan prior to surgery, and 12 months after surgery. PET scanning is optional and for research purposes only, which will be disclosed to all participants. PET scans of pediatric patients with anorexia who received NAcc-DBS has demonstrated the reversal of metabolic abnormalities, such as hypermetabolism in the frontal lobes, hippocampus, and lentiform nucleus [57]. The FDG-PET of participants in the present study may similarly demonstrate changes of metabolism in the brain following NAcc-DBS.

### Follow-up

Evaluation and assessments occur at approximately 22 time points from pre- to 1-year post-surgery (Table 3). Participants are followed weekly after surgery by the medical team between weeks 0–6, and monthly after 8 weeks. There are two required MRI scans that fall under the standard of care, two optional FDG-PET scans, and 3 possible 2-week periods of actigraph recordings. Surveys for the primary clinical outcomes and secondary outcomes are administered once prior to the surgery and four times following surgery. DBS programming starts at approximately 4–6 weeks and reassessment and stimulation changes are planned for weeks 12, 26, and 52 unless there are adverse effects, for which there will be additional programming visits. Ongoing follow-up for safety and DBS programming will occur at HSC until participants turn 18 years old, at which point they will be transitioned to adult care.

### Data management and statistical analysis

Patient information will be kept strictly confidential at all times, and all files related to this study will be password protected and kept on a password protected computer. Patient and caregivers will complete surveys and questionnaires online via a secure email link or on paper. The research team will check the content and completion of the forms monthly. All data will be kept for 25 years following the conclusion of the trial.

This is not a blinded study and all investigators and patients are aware of treatment. There is not blinded stimulation-off or stimulation-on periods after DBS implantation. Recruitment rates will be calculated monthly; the secondary feasibility and safety outcomes would be reported as proportions. Change in SIB and adverse effects will be analyzed longitudinally using comparative statistics. Repeated measures ANOVA and mixed-effects linear regression are used for analysis of clinical measures and changes over the course of 12 months. All efforts are made to reduce missing data with retrospective review and multiple imputation will be employed for unfound data.

## Discussion

The current paper presents a single-cohort, open-label, non-randomized, prospective pilot trial for the first controlled study of DBS of the NAcc for severe, refractory SIB in children and youth with ASD. This study is important to understand feasibility, safety, and treatment response. It is critical to establish feasibility of DBS for this novel indication and vulnerable patient population.

The rapid expansion of DBS indications in adult pathologies has allowed for treatment of many psychiatric diseases [58–60]. It is expected that the adaptation of DBS for children will be feasible for SIB. The early treatment of SIB in childhood could potentially be beneficial during critical windows of development such as in the adolescent or young adult years. The largest feasibility challenge of this study will be recruitment of vulnerable participants. This is mitigated by a multi-disciplinary team who are familiar with the patient population and the trial to allow for multiple sources of referral. Furthermore, results of a pilot trial should greatly improve recruitment in a future definitive trial. The use of multiple clinical measurement tools may help determine the optional composite measure of responsiveness of SIB to DBS. Although the quantitative data may not be rigorous enough to calculate a minimally clinical important difference, an estimated effect size may help determine sample size. Careful measurement and analysis of feasibility in this unique trial will be critical in the design of future research.

SIB has a high prevalence in several genetic disorders, such as Lesch-Nyhan disease and Fragile X syndrome, suggesting the pathophysiology of SIB may at least in part be rooted in neurobiological as well as neurodevelopmental etiologies [6]. Conversely, environmental triggers, such as novel sensory stimuli or changes in routine, often trigger the onset of episodes of SIB [61]. In patients with ASD, SIB frequency has a direct correlation with ASD severity [62]. The phenotype of SIB in patients with ASD shares similar features to phenotypic behaviors in other psychiatric conditions affecting children and adults, including OCD, attention-deficit hyperactivity disorder, and Tourette’s syndrome [63]. These conditions may be comorbid and share a common behavioral phenotype characterized by repetition, rigidity, invariance, and sometimes inappropriateness [38, 63]. Improved understanding of neural circuitry and effective DBS treatment options for one indication may enrich the understanding of the related diagnoses and their neurobiological pathophysiology.

DBS is a minimally invasive surgical option for patients with refractory SIB. It is reversible, adjustable and has a well-tolerated safety profile. Although increasingly offered to children for expanding indications, it is often considered an experimental therapy in this context. A proposed ethical framework to guide the conduct of DBS in children has been proposed, to which this study adheres [64]. The principles of this framework include viewing outcomes in a developmentally relevant context, cautiously applying adult data, and weighing the timing of the procedure [64].

Adult data and experience regarding DBS are not directly applicable to children. As one example, most children will have surgery under general anesthesia, which may preclude microelectrode recordings. Second, stimulation settings are often adopted from adult literature, yet long-term effects for children are not yet fully understood [23]. Third, indications and decisions regarding timing for intervention are complicated in children as treatment decisions are made within the context of childhood development and disease natural history. For example, the natural history of Tourette’s syndrome is often to remit as children develop and therefore the indications for DBS in children are as-of-yet unknown [65, 66]. Finally, complications including infection, lead migration or wire fractures are probably higher in children [21] and side effects of DBS on the developing brain require further study. Moreover, these hardware-related adverse events may manifest differently in children and youth with ASD and repetitive or compulsive SIB, relative to other pediatric cohorts.

## Strengths and limitations

This is a prospective, pilot trial of six patients and analyses of clinical outcomes will be limited by the small cohort. The results of this trial should inform larger clinical trials of multiple institutions. Furthermore, there is a lack of consensus regarding the ideal clinical assessment tool to measure the severity and frequency of SIB events. The use of the CYBOCS-ASD, ISAS and the RBS-R questionnaires incorporates several aspects of SIB such as repetitive movements, degree of harm, and impact on functional activities.

## Conclusion

This trial has primary objectives to assess the feasibility and safety of DBS for SIB in children with ASD. Clinical outcomes are composed of the change in CYBOCS-ASD, ISAS and the RBS-R questionnaires. Additional outcomes include measures of participant and caregiver quality of life, actigraph measurements, PET changes, and electrophysiologic data. These data will inform the design and conduct of future investigations in this vulnerable population to deliver novel effective therapies.

## Data Availability

The datasets generated and/or analyzed during the current study are not publicly available due to patient privacy given the small cohort but are available from the corresponding author on reasonable request.

## Abbreviations

ALIC: Anterior limb of internal capsule
ASD: Autism spectrum disorder
CGI-S: Clinical Global Impairment-Severity
CT: Computed tomography
CYBOCS-ASD: Children’s Yale-Brown Obsessive-Compulsive Scale in Autism Spectrum Disorder
DBS: Deep brain stimulation
DSM-V: Diagnostic and Statistical Manual of Mental Disorders, 5^th^ edition
FDG: Flurodeoxyglucose
GAF: Global assessment of functioning
HAS: Hamilton Anxiety Scale
HDS: Hamilton Depression Scale
HSC: Hospital for Sick Children
ISAS: Inventory of Statements about Self-Injury
JHMRS: John’s Hopkins motor stereotypy rating scale
K-ARS: Korean ADHD Rating Scale
MRI: Magnetic resonance image
NAcc: Nucleus accumbens
OAS: Overt Aggression Scale
OCD: Obsessive-compulsive disorder
PET: Positron emission tomography
RBS-R: Repetitive Behavioral Scale-Revised
SCQ: Social Communication Questionnaire
SIB: self-injurious behavior
SRS: Social Responsiveness Scale
Y-BOCS: Yale-Brown Obsessive-Compulsive Scale
